# Genome-wide *piggyBac* transposon mediated screening reveals genes related to reprogramming

**DOI:** 10.1007/s13238-016-0332-z

**Published:** 2016-10-19

**Authors:** Xi Zhang, Xinglin Wei, Yuanyuan Wu, Yuzhe Wang, Cheng Tan, Xiaoxiang Hu, Ning Li, Mario R. Capecchi, Sen Wu

**Affiliations:** 1 State Key Laboratory of Agrobiotechnology, College of Biological Sciences, China Agricultural University, Beijing, 100193 China; 2 Department of Human Genetics, University of Utah School of Medicine, Salt Lake City, UT 84112 USA

**Dear Editor**,

iPSCs are typically derived using four transcription factors Oct4 (also known as Pou5f1), Klf4, Sox2 and c-Myc (OKSM) (Takahashi and Yamanaka, 2006). Major directions in this field are focused on understanding the reprogramming mechanism, optimizing reprogramming methods to improve iPS cell (iPSC) quality, and applications of the cells for regenerative medicine. Over the past few years, great efforts have been put into finding novel genes involved in the reprogramming process. Indeed, increasing numbers of factors are found to play important roles in improving reprogramming efficiency (Theunissen and Jaenisch, 2014). However, it is still unclear how many more genes are implicated in this process, and methods are needed to systematically evaluate each gene’s relevance to reprogramming.

Phenotype-driven genetic screens with loss-of function and gain-of function methods have been successfully used to discover reprogramming factors. DNA transposons, RNA interference and CRISPR/Cas9-based systems have been powerful tools for loss-of function screening. A series of reprogramming factors have been identified with these loss-of-function methods (Ding et al., 2012; Kearns et al., 2014; Woltjen et al., 2016). However, the use of gain-of-function screening in reprogramming has been very limited. cDNA libraries, cell extract of pluripotent stem cells and high-throughput small molecule screens have also been exploited to find pluripotency genes (Abujarour et al., 2010; Singhal et al., 2010; Ying et al., 2008). These methods can only examine several genes at a time. A gain-of-function screen on the whole genome-scale would be very useful for saturation screens of reprogramming factors.

In this study, we aimed to systematically identify genes that participate in cellular reprogramming by establishing a gain-of-function screening strategy with PB vectors combined with the next-generation sequencing (NGS) and define each gene’s relevance to reprogramming.

The *piggyBac* transposon (PB) has been demonstrated to be an efficient mutagenesis tool in mammalian cells and mice (Ding et al., 2005; Wu et al., 2007) Compared with the commonly used retroviral vectors, PB transpositions in mammalian cells are more efficient and random (Wang et al., 2008). Therefore, we constructed a PB screening vector pFind1 that enabled a large-scale functional screening strategy for genes related to reprogramming (Fig. 1A and Fig. S1A). Although pFind1 is designed as a dual function vector, when used in cultured diploid cells, it primarily functions as a gain-of-function vector. To determine an efficient screening scheme, we first aimed to find a practical combination of reprogramming factors that alone generate no or minimal number of iPSC clones, but pFind1 addition can significantly increase colony numbers. We began by co-transfecting our cells with OK (Oct4 and Klf4) or OS (Oct4 and Sox2) combination (Fig. S1B), since OK or OS combinations alone rarely induced MEFs into iPSCs (Takahashi and Yamanaka, 2006). Neither OS + pFind1 nor OK + pFind1 showed significant difference of reprogramming efficiency compared with OK or OS alone (Fig. 1B).

**Figure 1:**
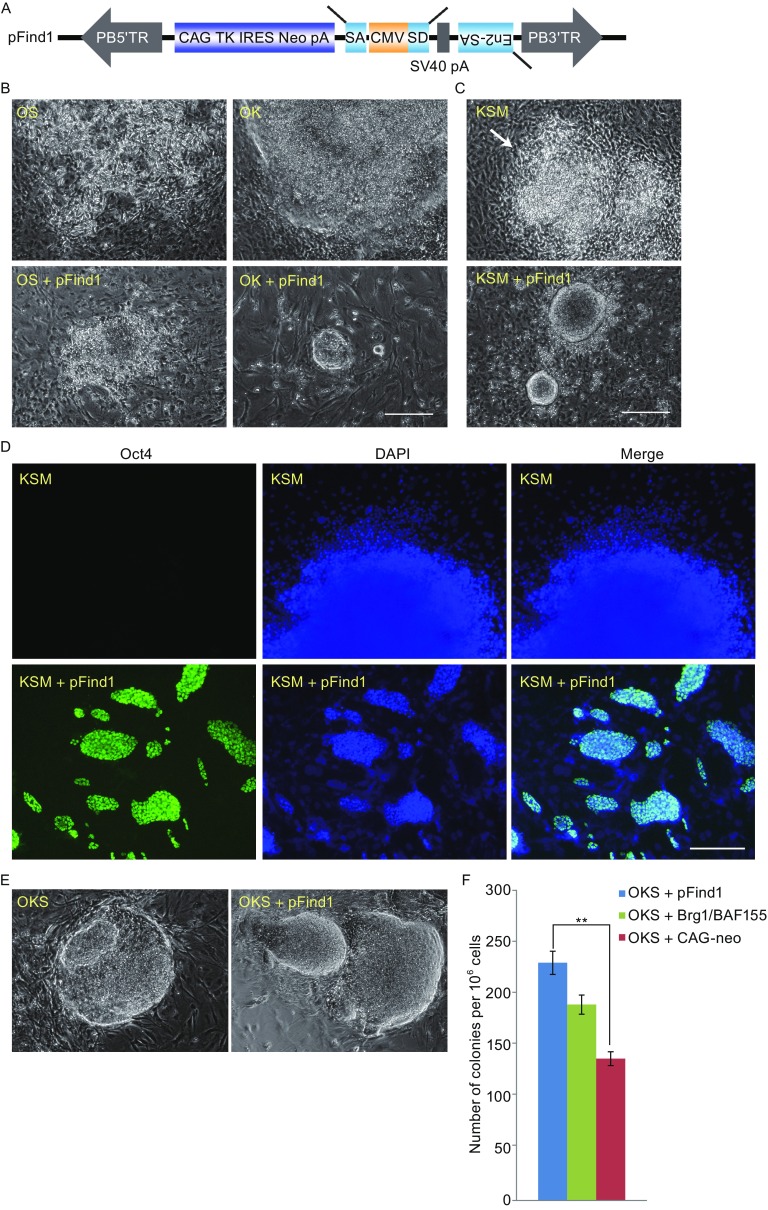

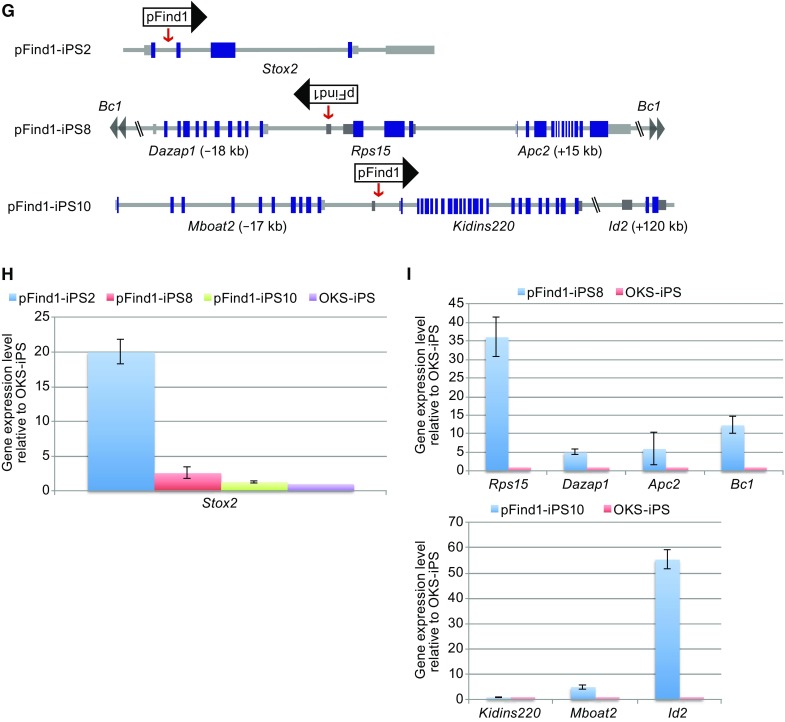
**The clonal screening method used to discover pluripotency genes on a small-scale**. (A) Diagram of *PB* screening vector pFind1 that contained a Cytomegalovirus (CMV) enhancer/promoter, a neomycin (G418) resistant gene and gene-trap cassette. (B) Morphology of cells induced by two transcription factors (OK/OS) with or without pFind1. Scale bar = 2 mm. (C and D) The iPSC clones generated by KSM and pFind1 express pluripotency markers. pFind1-iPSCs cultured on ES medium and harvested on 14 dpt were positive for Oct4. Scale bars = 1 mm. (E) Morphology of cells induced by OKS with or without pFind1. Scale bar = 2 mm. (F) The insertion of pFind1 resulted in an increase of iPSC colonies induced from Oct4-GFP MEFs. CAG-neo means an empty PB vector that only contained CAG-*neo*. All data are represented as mean ± S.D., *n* = 3. A Student t-test was used for statistics. Asterisks indicate statistical significance: **P < 0.01, *P* = 0.008. (G) Analysis of pFind1 integration sites in three clones shown by arrows. The insertion sites were identified by inverse-PCR and sequencing analysis. Blue boxes indicate coding exons; white boxes indicate non-coding exons. The red arrows show pFind1 insertion sites; the black arrows show the orientation of pFind1 at integration sites. The data in brackets indicate the distance from primers for qRT-PCR to pFind1 insertion sites. We set pFind1 insertion sites as origin. Positive number represented downstream of insertion sites; Negative number represented upstream of insertion sites. (H) Quantitative reverse transcription PCR (qRT–PCR) assay was used to evaluate the gene expression profile of pFind1 insertion neighboring genes in pFind1-iPSC lines and control OKS-iPSC lines. Transcript levels were normalized against expression of internal control (*GAPDH*). All data are represented as mean ± S.D., *n* = 3. *p*(pFind1-iPS2) = 0.004; *P* (pFind1-iPS8) = 0.041; *P*(pFind1-iPS10) = 0.579. (I) qRT–PCR assay was used to evaluate the expression level of genes that are located at different distances away from pFind1 insertion sites in pFind1-iPSC lines and control OKS-iPSC lines. Transcript levels were normalized against expression of internal control (*GAPDH*). All data are represented as mean ± S.D., *n* = 3. *P*(Rps15) = 0.005; *P*(Dazap1) = 0.012; *P*(Apc2) = 0.023; *P*(Bc1) = 0.002; *P*(Kidins220) = 0.859; *P*(Mboat2) = 0.021; *P*(Id2) = 0.001.

We further tested pFind1 and three-factor combinations KSM (Klf4, Sox2 and c-Myc) or OKS (Oct4, Klf4 and Sox2) (Fig. S1B). KSM did not reprogram fibroblasts into iPSC colonies, and only partially reprogrammed cell clusters could be found (Fig. 1C) (Shu et al., 2013; Takahashi and Yamanaka, 2006). However, when both KSM and pFind1 were introduced into MEFs, we detected colonies that exhibited similar morphology to mouse ES cells and expressed alkaline phosphatase (AP) (Fig. S1C) and pluripotency marker Oct4 (Fig. 1D), demonstrating that insertion of pFind1 promoted cellular reprogramming. Five colonies were picked to identify genomic insertion sites. Among the genes identified, *Smad3* has been reported to play an important role in maintaining mouse ESC stability (Li et al., 2013). This result from KSM + pFind1 screening indicates that our PB vectors can be used to find factors that improve quality of iPSCs. Because of the low reprogramming efficiency (0.001‰), KSM plus pFind1 could only provide very few colonies for high-throughput screening. We next tested alternative screening scheme with OKS and pFind1 combination.

To test the reliability and validity of OKS/pFind1 strategy, we used a reporter cell line Oct4-GFP MEFs harboring a stably integrated green fluorescent protein (GFP) cassette driven by an Oct4 promoter and enhancer to monitor iPSC generation. iPSC clones derived from OKS alone or OKS + pFind1 (Fig. 1E) showed similar morphology. We next estimated whether the addition of pFind1 affected reprogramming efficiency. As a positive control, we supplied Brg1/Baf155 chromatin remodeling complex proteins that had previously been reported to increase the number of GFP^+^ colonies up to 4-fold (Singhal et al., 2010). An empty vector was used as a blank control. On these blank control plates, colonies first appeared around 10 days post transfection (dpt). In contrast, iPSC colonies appeared a few days earlier in the experiment plates that were transfected with OKS, pFind1 and pCAG-PBase. When GFP^+^ colonies were counted at 14 dpt, we found that pFind1 exhibited a clear increase in reprogramming efficiency (Fig. 1F).

We next manually picked 300 iPSC clones from the experiment plates at 8 dpt in order to catch pFind1-facilitated colonies. All these clones were expanded for further analysis. Through inverse-PCR followed by sequencing analysis, we identified pFind1 genomic insertion sites (Fig. S1D). In these ~300 iPSC clones we identified 213 genes including known pluripotency genes, such as Nr5a1, Klf5, mir205 (Table S1). To detect whether pFind1 can modulate expression of genes adjacent to insertion sites, we examined three random iPSC clones (pFind1-iPS2, pFind1-iPS8 and pFind1-iPS10) derived with OKS and pFind1 in details. We identified pFind1 insertion sites (Fig. 1G) and analyzed expression of neighboring genes. Increased expression of *Stox2* (a gene encoding Storkhead Box 2 protein) was significant in pFind1-iPS2 with pFind1 insertion (Fig. 1H), compared with other clones that have completely different integration sites (not adjacent integration) and iPSC clones induced with OKS only (OKS-iPS). These results indicated that the pFind1 insertions activated neighboring genes at the transcription level. Further, we found that the *Id2* gene 120 kb away from insertion site was also up-regulated by pFind1 (Fig. 1I), suggesting that the effect range of pFind1 could reach 120 kb. Together, these results demonstrate that pFind1 vector can be used for large-scale screening for reprogramming related genes.

Because the identification of pFind1 insertion sites in single colonies is tedious and rate limiting, we designed a high-throughput method to detect pFind1 insertion sites with NGS (Fig. S2A). We collected nearly 500,000 iPSC colonies derived with pFind1 and OKS. To detect a potential genomic insertion bias of pFind1, we concurrently performed a control with the pFind1 vector alone. pFind1 integration sites were mapped through a modified inverse PCR protocol that created fragments compatible with high-throughput sequencing.

After filtering and processing the original deep sequencing data, we aligned the sequences that were adjacent to pFind1 insertion sites to UCSC database (http://genome.ucsc.edu). pFind1 insertions were distributed among all chromosomes (Fig. S2B). We calculated frequencies of the pFind1 insertion in each chromosome, which were used to create distribution histograms of insertion frequencies for 19 mouse autosomes and 2 sex chromosomes (Figs. 2A and S3A). Through statistical analysis of genes located around pFind1 integration sites, a ranking list of 12,634 genes was obtained by counting pFind1 hit times around each gene (Table S2). In the control experiment of MEFs transfected with pFind1 only, pFind1 insertions showed dramatically different patterns (Fig. S3B), suggesting that the different pFind1 insertions in our screening experiment resulted from pFind1 enhanced reprogramming.

**Figure 2:**
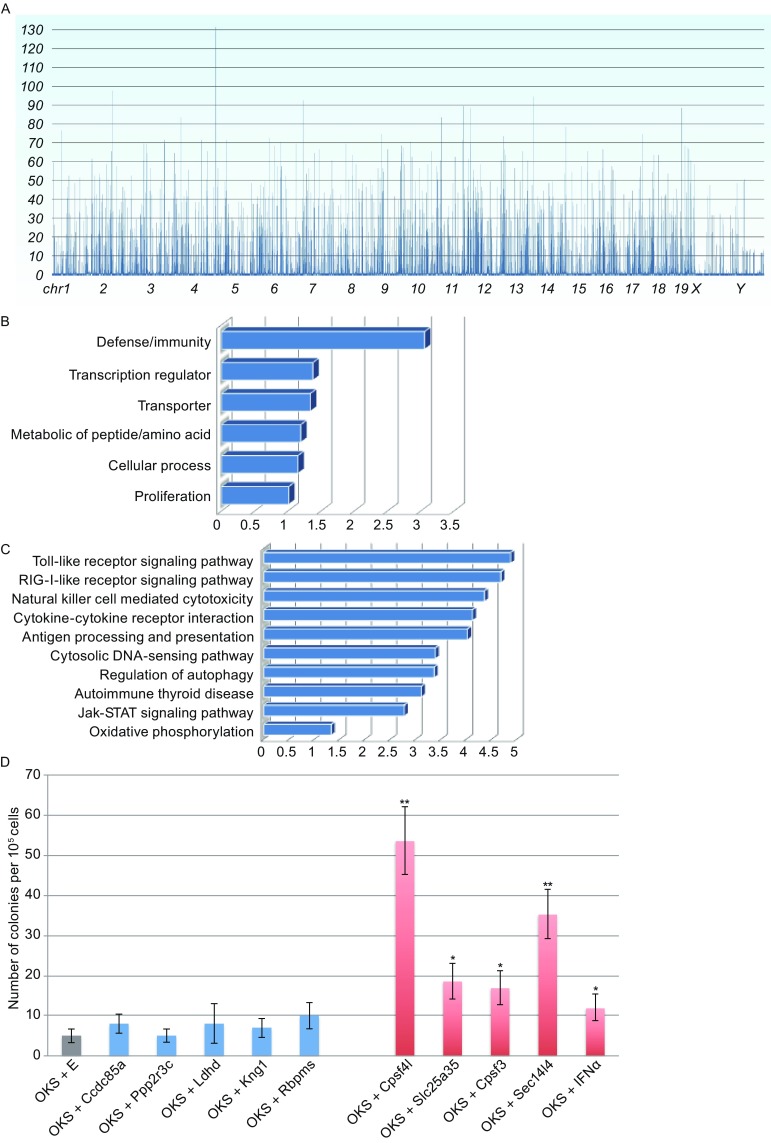
**A large-scale screen for reprogramming regulators using OKS/pFind1 and identification of gene’s capacity to cellular reprogramming**. (A) The distribution diagram of 21 chromosomes during the generation of iPSCs. (B) Gene ontology (GO) enrichment analysis of top-ranked 1000 genes activated by pFind1. (C) KEGG pathway analysis of top-ranked 1000 genes activated by pFind1. (D) Identification of gene’s capacity to cellular reprogramming. Low ranking genes are labeled in blue; high ranking genes are labeled in red. E is a negative control vector that contains green fluorescent protein (GFP) cassette. All data are represented as mean ± S.D., *n* = 3. Student t-tests were used for statistics. Asterisks indicate statistical significance: * *P* < 0.05, **P < 0.01. *P*(Cpsf4l) = 0.002; *P*(Slc25a35) = 0.030; *P*(Cpsf3) = 0.038; *P*(Sec14l4) = 0.007; *P*(IFNα) = 0.031

Depending on the location of pFind1 insertion sites, dual function design of pFind1 can result in either loss-of-function or gain-of-function of the gene. We isolated genes that were disrupted (Table S3) and genes that were up-regulated (Table S4). Since our sequencing results indicated that most pFind1 insertions are located in intergenic regions, we mostly focused on gain-of-function aspect in the screen. We ranked these genes that were up-regulated by pFind1 via calculating pFind1 insertion frequency around each gene. To get a global picture of these genes, functional annotations were performed using GO (Gene Ontology) analysis (Fig. 2B) and KEGG (Kyoto encyclopedia of genes and genomes) analysis (Fig. 2C). Many enriched genes were related to well known self-renewal signaling pathways such as Jak-STAT signaling pathway (Raz et al., 1999). The most significantly enriched genes were related to innate immunity pathways. These results suggest innate immunity pathways may be involved in reprogramming.

To further validate our gene ranking list, by comparing with a previously published list of genes that could potentially increase iPSC quality (Wu et al., 2014), we selected 10 genes covering different segments in Table S4 to examine their reprogramming capability. We electroporated each candidate gene’s overexpression vector along with OKS into MEFs and calculated efficiency of iPSC generation. All these genes can promote generation of iPSCs to various extents with a trend that high ranking genes markedly enhanced iPSC generation whereas low ranking genes only had slight effect, although there was no strict correlation (Fig. 2D). These validation results suggest that our gene ranking list from the screen can indeed provide a meaningful reference to evaluate each gene’s reprogramming ability in the future.

In the current study, we developed a new screening method for large-scale screening reprogramming-related genes based on PB transposon technology and NGS. We illustrated the utility of this method by rapid and systematic screening of mouse genome for reprogramming factors. Several features of our method are worth mentioning. First, the use of PB transposon mutagens allowed us to quickly generate a large collection of iPSCs with different insertions covering the entire mouse genome. Second, the gain-of-function design of pFind1 enabled us to perform a genetic screen for reprogramming factors in a sensitized background. Third, combined with deep sequencing and bioinformatic analysis, the distribution of pFind1 insertions can be easily identified, allowing one to address gene function on a large scale. The frequency of PB insertion in our study is not an absolute measure of a gene’s reprogramming ability, but it can very well provide a good starting point for further assessment of each gene’s relevance to reprogramming. This method should have broader applications to reveal genes participating in other biological processes.

## Electronic supplementary material

Below is the link to the electronic supplementary material.

Supplementary material 1 (PDF 264 kb)

Supplementary material 2 (XLS 58 kb)

Supplementary material 3 (XLS 2178 kb)

Supplementary material 4 (XLS 287 kb)

Supplementary material 5 (XLS 1605 kb)

Supplementary material 6 (DOCX 14 kb)

Supplementary material 7 (DOCX 16 kb)
