# Supplementary material for: Genome-wide piggyBac transposon mediated screening reveals genes related to reprogramming
Source: Protein Cell. 2016 Oct 19;8(2):134–9. doi: 10.1007/s13238-016-0332-z (PMC5291772; doi:10.1007/s13238-016-0332-z)
Supplement: Supplementary file 1 — Supplementary material 1 (PDF 264 kb) [file 13238_2016_332_MOESM1_ESM.pdf]

Supplemental Information

Materials and Methods

Plasmid Construction

The pFind1 vector containing 5' and 3' PB terminal NA repeats was derived from ZG-s (Wu et al., 2007).The internal ribosomal entry site (IRES) and splice acceptor (SA) were also from ZG-s. The splice donor (SD) and En2-SA were widely used gene-trap cassette (Dupuy et al., 2005).

Cell Culture

MEFs from strain 129Sv were derived from embryos at embryonic day 13.5 (E13.5) and cultured in DMEM containing 15% (vol/vol) FBS (Life). MEFs from the Oct4–GFP transgenic mouse line [Jax Mice strain name: B6; CBA-Tg (Pou5f1-EGFP) 2Mnn/J; stock no. 004654] were isolated following the protocol previously described (Wu et al., 2014). Mouse iPSCs were maintained on feeder layers and cultured in ES medium containing DMEM with 15% (vol/vol) FBS, 500 units/mL Lif (Millipore; ESG1107), 0.1 mM 2-mercaptoethanol and 50 units/50 mg/mL penicillin/streptomycin (Pen-Strep). Mouse iPSCs were maintain in 2i medium that was prepared by mixing 500 mL of DMEM/F12 medium (Invitrogen; 10565-042), 500 mL of Neurobasal medium (Invitrogen; 21103-049), 10 mL of B27 supplement (Invitrogen; 17504-044), 5 mL of N2 supplement (Invitrogen; 17502-048), 5 mL of 100 × Pen-Strep (Invitrogen; 15070-063), 500 units/mL human Lif (Millipore; LIF1010), 0.1 mM 2-mercaptoethanol, 1 μM MEK inhibitor PD0325901 (Selleck), and 3 μM GSK3β inhibitor CHIR99021 (Selleck) (Buehr et al., 2008; Ying et al., 2008).

MEF Reprogramming

3×10^6 MEFs were electroporated with 3 μg OKS, 5 μg pFind1, and 3 μg pCAG-PBase plasmids using the Lonza Amaxa Nucleofector, program A-024. Electroporated cells were reseeded on feeder layers and transferred in ES medium. At 20–24 h after transfection, we applied drug selection at a concentration of 1 μg/mL puromycin (Sigma). Puromycin selection lasted for 5 d. Colonies were manually picked at 8 dpt.

Inverse PCR and Sequencing Analysis

Genomic DNA (gDNA) was extracted using the protocol described previously (Wu et al., 2008) and an adapted inverse PCR was employed to identify insertion sites. *Msp*I digests gDNA were purified with Qiagen columns and self-ligated to serve as the template for PCR. We performed two rounds inverse PCR to identify pFind1 insertions for sequencing analysis of single clones. PB152 and PB153 were primers used to recover the flanking sequence of the 5' of pFind1. PB36 and PB149 were primers used to recover the flanking sequence of the right side of 3' of pFind1. In order to reduce the influence of PCR bias on next-generation sequencing, one round of inverse PCR was carried out to identify pFind1 junction sites in 500,000 colonies. The primers were PB152 and PB36. Primer sequences are listed in Supplementary Table S5. The Roche Expand long template PCR kit was used for all inverse PCR reactions. The PCR conditions followed the protocol previously described (Wu et al., 2007). PCR products were purified with Qiagen columns for subsequent sequencing. Sequencing results were analyzed with NCBI BLAST (www.ncbi.nlm.nih.gov).

Quantitative RT-PCR

Total RNA was isolated using RNeasy Mini Kit (Qiagen). First-strand cDNA was synthesized using QuantiTect Reverse Transcription Kit (Qiagen) according to manufacturer's protocol. Primer sequences used for RT-PCR were given in

Supplementary Table S6. For quantitative RT-PCR, LightCycler 480 SYBR Green I Master (Roche) was used.

Next Generation Sequencing

To generate a library for ion torrent sequencing 5 µg PCR products were sheared into DNA fragments by ultrasonic fragmentation. Then end-repair reaction was carried out. Thereafter, Ion Torrent compatible adapters were ligated to the fragments. The adapter sequences are listed in Supplementary Table S5. For optimal sequencing results, size-select a DNA library with a mean size of 180–210 bp, with a distribution of approximately ±20 bp around the mean. This was followed by nick-translation and 3–5 cycles of PCR to amplify the sequencing library. The number of cycles was minimized to avoid over amplification and bias. Then we determined the library dilution required for template preparation and assessed the library size distribution. Finally, we loaded the sample on the Ion 318™ chip and performed the sequencing according to the manufacturer's instruction.

References

Buehr, M., Meek, S., Blair, K., Yang, J., Ure, J., Silva, J., McLay, R., Hall, J., Ying, Q.L., and Smith, A. (2008). Capture of authentic embryonic stem cells from rat blastocysts. Cell 135, 1287-1298.

Dupuy, A.J., Akagi, K., Largaespada, D.A., Copeland, N.G., and Jenkins, N.A. (2005). Mammalian mutagenesis using a highly mobile somatic Sleeping Beauty transposon system. Nature 436, 221-226.

Wu, S., Wu, Y., Zhang, X., and Capecchi, M.R. (2014). Efficient germ-line transmission obtained with transgene-free induced pluripotent stem cells. Proc. Natl. Acad. Sci. U.S.A. 111, 10678-10683.

Wu, S., Ying, G., Wu, Q., and Capecchi, M.R. (2007). Toward simpler and faster genome-wide mutagenesis in mice. Nat. Genet. 39, 922-930.

Wu, S., Ying, G., Wu, Q., and Capecchi, M.R. (2008). A protocol for constructing gene targeting vectors: generating knockout mice for the cadherin family and beyond. Nat. Protoc. 3, 1056-1076.

Ying, Q.L., Wray, J., Nichols, J., Batlle-Morera, L., Doble, B., Woodgett, J., Cohen, P., and Smith, A. (2008). The ground state of embryonic stem cell self-renewal. Nature 453, 519-523.

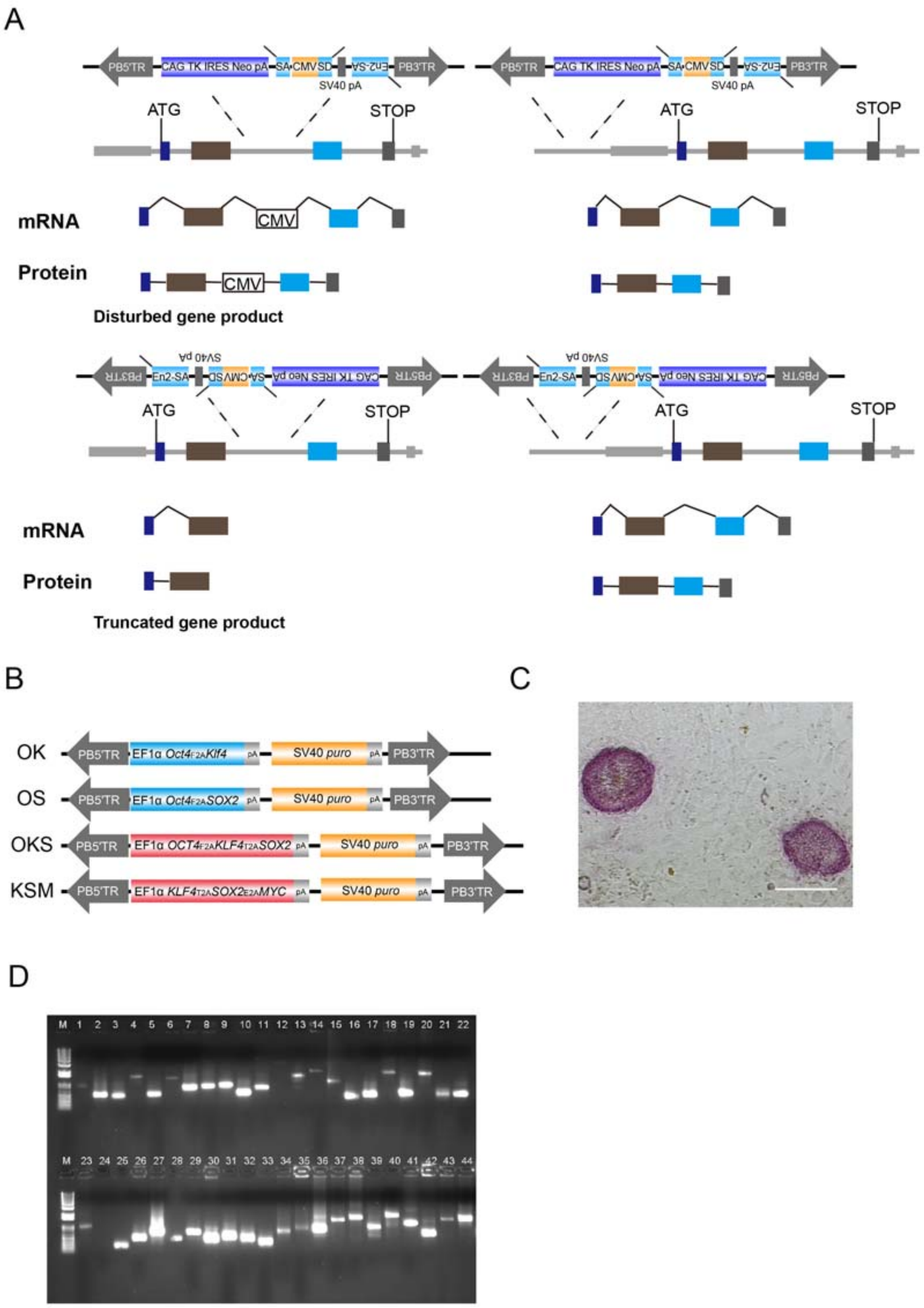

Figure S1. pFind1 mediated small scale screening.

(A) Schematic presentation of pFind1 insert in different region at sense or antisense orientation. pFind1 is designed as a dual function vector for both gain-of-function and

loss-of-function screening. When pFind1 is integrated in either orientation within a gene, the endogenous mRNA splicing is hijacked, due to the presence of exogenous two splice acceptors (SA), hence, the corresponding protein product is truncated. Since pFind1 contains Cytomegalovirus (CMV) promoter/enhancer and a splice donor (SD), it can also function to activate gene expression.

(B) Maps of PB vectors OK, OS, KSM and OKS.

(C) Morphology of cells induced by KSM with or without pFind1. The arrow pointed cell cluster generated by KSM. Scale bar = 2 mm.

(D) Inverse PCR analysis used for the clonal method. Each lane corresponds to one clone; each band corresponds to single pFind1 insertions. A representative gel electrophoresis picture shows partial results.

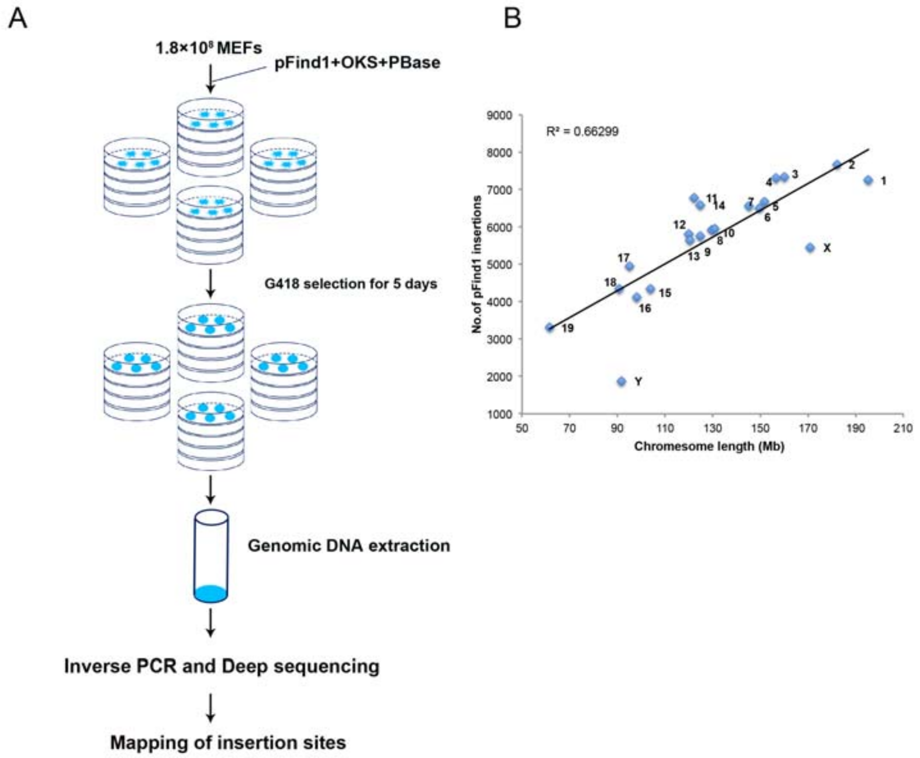

Figure S2. The establishment of large-scale screen for reprogramming regulators using OKS/pFind1.

(A) Genome-wide screen strategy for identification of genes associated with reprograming.

(B) pFind1 insertions dispersed randomly across the genome.

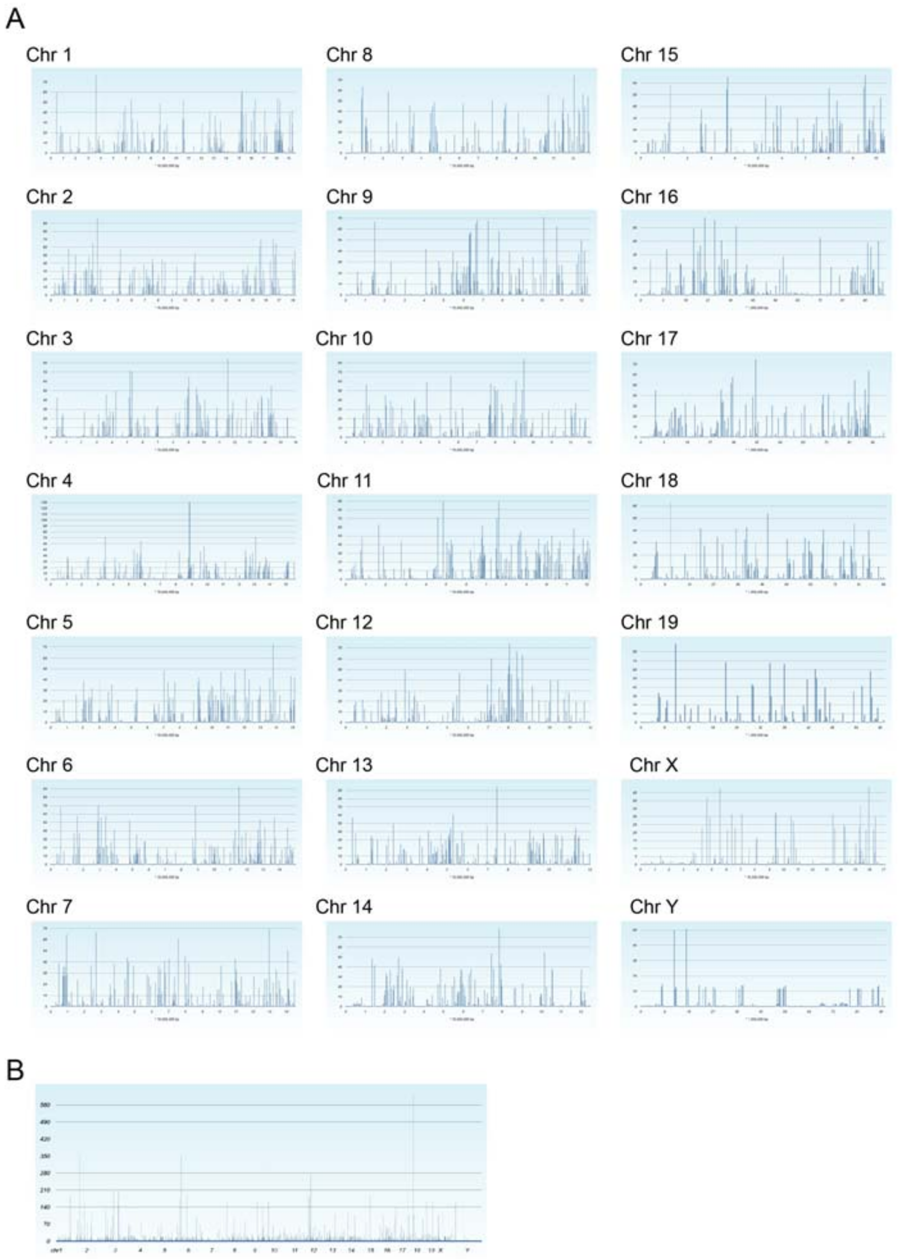

Figure S3. Data analysis of the large-scale screen strategy.

(A) The frequency distribution diagrams of pFind1 junction sites for 19 mouse autosomes and 2 sex chromosomes.

(B) Distribution of pFind1 insertions in MEFs was used as a control for large-scale screening experiment.

Table S1. Genes identified from 300 manually-picked iPSC clones.

Table S2. The ranking list of ~ 12,000 genes based on pFind1 insertion frequency.

Table S3. The ranking list of genes whose product was destructed.

Table S4. The ranking list of genes activated by pFind1.

Table S5. Primers and adapters used in the study.

Table S6. Primers used for qRT-PCR.
