# Supplementary material for: Genome-wide piggyBac transposon mediated screening reveals genes related to reprogramming
Source: Protein Cell. 2016 Oct 19;8(2):134–9. doi: 10.1007/s13238-016-0332-z (PMC5291772; doi:10.1007/s13238-016-0332-z)
Supplement: Supplementary file 6 — Supplementary material 6 (DOCX 14 kb) [file 13238_2016_332_MOESM6_ESM.docx]

**Table S5. Primers and adapters used in the study**

|  | **primer name** | **5'-primer sequence-3'** |
| --- | --- | --- |
| Inverse PCR Primers for pFind1 colonies 1st Round | PB36inv5F | CAGCGACACCCACCGCACCGACAAGCACGC |
|  | PB152 | CCGCAAGCCACGCAACGCGGAACCC |
| Inverse PCR Primers for pFind1 colonies 2nd Round | PB149 | GCGACCGAGACGCCCCAAACGCAC |
|  | PB153 | GGGCCACGAACCAACGACCCCGCAA |
| Ion Torrent adapters for next generation sequencing | A adapter | CCATCTCATCCCTGCGTGTCTCCGACTCAG |
|  | P1 adapter | CCTCTCTATGGGCAGTCGGTGAT |
