# Supplementary material for: Genome-wide piggyBac transposon mediated screening reveals genes related to reprogramming
Source: Protein Cell. 2016 Oct 19;8(2):134–9. doi: 10.1007/s13238-016-0332-z (PMC5291772; doi:10.1007/s13238-016-0332-z)
Supplement: Supplementary file 7 — Supplementary material 7 (DOCX 16 kb) [file 13238_2016_332_MOESM7_ESM.docx]

**Table S6. Primers used for qRT-PCR**

| **Gene** | **Primer Name** | **Primer Sequence** |
| --- | --- | --- |
| Stox2 | zhx142-Stox2 | GAAGTCTCGGTCACACAGCAAGAC |
|  | zhx143-Stox2 | TGGTTAGAGGGTCGCAAAATTCAT |
| Rps15 | zhx148-Rps15 | GTGTACAACGGCAAGACCTTCAAC |
|  | zhx149-Rps15 | CGGGTTTGTAGGTGATGGAGAAC |
| Dazap1 | zhx150-Dazap1 | GACGAAATCGGGAAGCTCTTTG |
|  | zhx151-Dazap1 | GATGACACAATCCACAACCTCACC |
| Apc2 | zhx152-Apc2 | ACAGAAACTCCTGTCCCAATCGAG |
|  | zhx153-Apc2 | TCATTCATAGCCCGACGGTATTCT |
| Bc1 | zhx154-Bc1 | TTAGCTCAGTGGTAGAGCGCTTG |
|  | zhx155-Bc1 | TTTTCCAGAGCTGAGGACCGAAC |
| Kidins220 | zhx162-Kidins220 | ATAAGCGGCAGATCTTCTCCACAC |
|  | zhx163-Kidins220 | TAGCTCATTCTCCTTCCCCCTTTC |
| Mboat2 | zhx164-Mboat2 | CCTCTCCATAAAACCGTCGTTCAC |
|  | zhx165-Mboat2 | TGAGACTTTTTCACAGGCAGCAAC |

| Id2 | zhx166-Id2 | GCACGTCATCGATTACATCTTGG |
| --- | --- | --- |
|  | zhx167-Id2 | GGTTCTGTCCAGGTCTCTGGTG |

| GAPDH | zhx188-mGAPDH | AAGGTCATCCCAGAGCTG |
| --- | --- | --- |
|  | zhx189-mGAPDH | CCTGCTTCACCACCTTCTTG |
